# Survival of Individuals Diagnosed With High‐Risk, Regional, and Metastatic Prostate Cancer by Culturally and Linguistically Diverse Status: A Retrospective Cohort Study Set in Victoria, Australia

**DOI:** 10.1002/cam4.71779

**Published:** 2026-04-14

**Authors:** Koku Sisay Tamirat, Nathan Papa, Jeremy Millar, Eli Ristevski, Michael James Leach

**Affiliations:** ^1^ School of Rural Health Monash University Warragul Victoria Australia; ^2^ School of Public Health and Preventive Medicine Monash University Melbourne Australia; ^3^ School of Translational Medicine Monash University Melbourne Australia; ^4^ Radiation Oncology Alfred Health Melbourne Australia; ^5^ School of Rural Health Monash University Bendigo Victoria Australia

**Keywords:** culturally and linguistically diverse backgrounds, mortality, prostate cancer, survival outcomes

## Abstract

**Background:**

This study aims to investigate survival outcomes among individuals with high‐risk, regional, and metastatic prostate cancer (PCa) by culturally and linguistically diverse (CALD) status in Victoria, Australia.

**Methods:**

Individuals with high‐risk, regional, and metastatic PCa (February 2009–February 2024) registered in the Victorian Prostate Cancer Outcomes Registry (PCOR‐Vic), with last follow‐up on 31 July 2024. CALD status was derived from study participants' country of birth and subgrouped into: mainly non‐English‐speaking countries (NESC) (CALD backgrounds), mainly English‐speaking countries (MESC) outside Australia (MESC‐born), and Australia (reference group). The primary outcomes, all‐cause mortality (ACM) and PCa‐specific mortality (PCSM), were determined through linkage of PCOR‐Vic to Victorian Cancer Registry death data. ACM and PCa‐specific survival were estimated using Kaplan–Meier curves and the cumulative incidence function. Weibull parametric survival and Fine‐Gray competing‐risks regression models, adjusted for sociodemographic and clinical variables, assessed the association of CALD status with ACM and PCSM.

**Results:**

Of 10,024 eligible participants, 32% died. The median survival time (death) was 116 months, and median follow‐up time (censoring) was 66 months. CALD individuals had lower 5‐year overall survival compared to Australian‐born individuals (66% vs. 71%). While unadjusted models suggested higher ACM among CALD individuals (adjusted hazard ratio [aHR] = 1.20, 95% confidence interval [CI] = 1.11–1.30), adjusted models showed lower ACM (aHR = 0.89, 95% CI = 0.82–0.97) and PCSM (adjusted subdistribution hazard [asHR] = 0.86, 95% CI = 0.76–0.96) than Australian‐born individuals. There were significant (*p*‐value < 0.05) interactions between CALD and age (older CALD individuals had higher hazard of ACM and PCSM) and CALD and PCa risk category (CALD individuals with regional PCa had higher hazard of ACM).

**Conclusions:**

While CALD individuals had lower overall survival and higher ACM than Australian‐born individuals, CALD individuals exhibited lower ACM and PCSM following adjustments. Interactions of age and PCa risk category with CALD influenced mortality. Contributing factors (e.g., late diagnosis) should be addressed to reduce disparities by CALD status.

## 1 Introduction

In Australia, prostate cancer (PCa) is the most common cause of male cancer mortality, accounting for 13% of cancer deaths among males during 2022 [1]. Five‐year relative survival from PCa has increased from 60% in 1987–1991 to 96% in 2013–2017 [2]. Australia is one of 25 countries with a PCa 5‐year survival rate ≥ 90% [3]. While PCa survival in Australia is generally improving and among the best worldwide, lower PCa survival persists for certain population subgroups. For example, non‐metropolitan residents, Aboriginal and Torres Strait Islander people, and socioeconomically disadvantaged individuals have relatively low PCa survival outcomes [4, 5, 6, 7].

Prostate cancer (PCa) survival may vary by culturally and linguistically diverse (CALD) status. The initial health advantages due to the healthy migrant effect (HME) among CALD and/or immigrant people tend to diminish over time, leading to similar or worse health outcomes than native‐born counterparts [8, 9]. This deterioration can be influenced by ageing, acculturation, and adopting unhealthy lifestyles [10]. There is also evidence suggesting that Australians from CALD backgrounds—such as immigrants and non‐native English speakers—may be medically underserved [9, 11]. The Australian Cancer Plan identified individuals from CALD backgrounds (hereafter termed ‘CALD individuals’) as a priority population at risk of poorer cancer outcomes [12]. Furthermore, the Australian ‘Optimal Care Pathway for Men with Prostate Cancer’ emphasises the importance of addressing CALD individuals' healthcare needs, to improve their survival outcomes [13]. In Australia, CALD individuals are more socioeconomically disadvantaged, have limited access to quality healthcare, and are less likely to participate in PCa screening and early detection programs than Australian‐born individuals [9, 14, 15, 16].

Factors influencing PCa survival in Australia include age, clinicopathological characteristics (e.g., National Comprehensive Cancer Network [NCCN] risk groups), and type of institutions delivering healthcare [5, 6]. Australian and international studies found that CALD individuals with PCa presented with significantly more high‐risk or advanced diseases, which carry a poorer prognosis [17, 18]. However, there is little data on PCa survival outcomes by CALD status in Australia, making resource allocation, progress monitoring, and data‐driven decision‐making difficult. Such data are increasingly important. The number of Australian residents born overseas increased from 4.4 million (23.0%) in 2000 to 7.7 million (29.5%) in 2022, while the number of people who spoke a main language other than English grew from 4.9 million (21.6%) in 2016 to 5.8 million (22.8%) in 2022 [19]. Improved understanding of PCa survival outcomes by CALD status may inform the design of culturally tailored interventions to achieve equitable survival outcomes for Australia's increasingly multicultural population.

We aimed to investigate all‐cause mortality (ACM) and PCa‐specific mortality (PCSM) by Culturally and linguistically diverse (CALD) status among individuals with high‐risk, regional, and metastatic PCa in Victoria, Australia. We hypothesised that mortality outcomes (ACM and PCSM) would vary by individuals’ CALD status, and that differences may depend on age and NCCN risk category at diagnosis.

## 2 Methods and Materials

### 2.1 Data Source

The Victorian Prostate Cancer Outcomes Registry (PCOR‐Vic) provided data on individuals diagnosed with PCa. This registry's recruitment and data collection processes have been described previously [20]. Briefly, PCOR‐Vic sends an opt‐out enrolment invitation to all individuals newly diagnosed with PCa and statutorily notified to the Victorian Cancer Registry (VCR). PCOR‐Vic receives patient data from contributing health services, which diagnose approximately 87% of PCa cases in Victoria, Australia. PCOR‐Vic collects sociodemographic, clinical, treatment, and outcome data to monitor, evaluate, and understand patterns of care and survival outcomes for individuals with PCa.

### 2.2 Study Design

A retrospective cohort study was conducted.

### 2.3 Study Population

**FIGURE 1:**
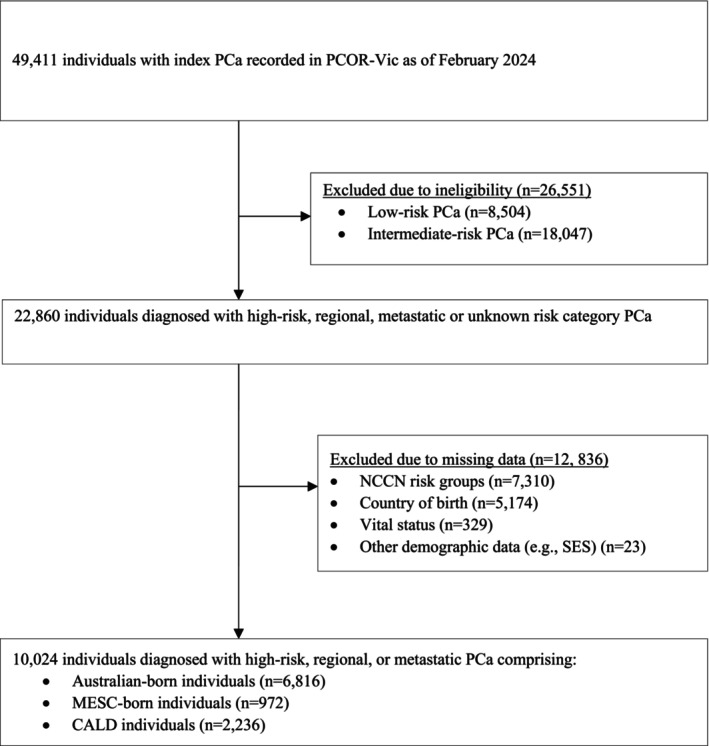
Flow diagram of PCOR‐Vic registrants excluded from the study population. CALD, culturally and linguistically diverse; MESC, mainly English‐speaking countries; NCCN, National comprehensive cancer network; PCa, prostate cancer; PCOR‐Vic, Victorian prostate cancer outcomes registry; SES, socioeconomic status.

We included PCOR‐Vic registrants diagnosed over February 2009–February 2024 with high‐risk, regional, or metastatic (T_any_N_0‐1_ M_0‐1_) PCa. Our study population was restricted to high‐risk, regional, and metastatic disease—priority risk categories potentially associated with worse outcomes or lower survival rates compared to low‐ and intermediate‐risk disease [21, 22]. In addition, for low‐ and intermediate‐risk disease, 5‐ and 10‐year survival rates approach 100%, with very few mortality expected events [23], posing methodological challenges such as unstable statistical estimates and reduced power to detect differences. Registrants were excluded if they were ineligible or, in line with complete‐case analysis, lacked data on one or more variables of interest (Figure 1).

### 2.4 Measures

#### 2.4.1 Exposures

The primary exposure variable was CALD status derived from country of birth. Our definition of CALD status aligns with prior Australian studies [24, 25] and the Australian Institute of Health and Welfare's guidance on reporting on the health status of Australians from CALD backgrounds [26]. Individuals were classified as born in: mainly non‐English‐speaking countries (NESC) (CALD backgrounds), mainly English‐speaking countries (MESC) outside Australia (MESC‐born), and Australia (reference group).

The Australian population is becoming more diverse over time. In 2022 ABS data, three in ten Australians were born overseas, and 22% spoke languages other than English at home, with the dominant immigrant countries of birth shifting from European to Asian origins [19]. Cognizant of these changes in migration trends, the Australian Bureau of Statistics (ABS) has developed a standard to define CALD using a minimum core set: country of birth, main language other than English spoken at home, proficiency in spoken English, and Indigenous status [27]. Detailed and disaggregated analysis by CALD‐defining indicators may help identify disparities and population groups at risk, and inform interventions to improve equitable cancer care and outcomes, as ‘one size does not fit all’ [26]. Despite setting these CALD‐defining indicators, there are considerable inconsistencies in how CALD status is defined and determined [25]. We defined and categorised CALD status into three categories, based on evidence and approaches from previous Australian studies [24, 25].

#### 2.4.2 Other Covariates

Sociodemographic characteristics included age at diagnosis (years) and calendar year‐of‐diagnosis (2009–2016, 2017–2019, and 2020–2024). The residential area was determined from postcodes using the 2019 Modified Monash (MM) Model, with the most frequent scores used in instances of multiple scores for one postcode [28]. Area‐level socioeconomic status (SES) was determined from postcodes using the 2016 Socio‐Economic Indexes for Areas‐Index of Relative Socio‐economic Disadvantage (SEIFA‐IRSD) quintiles [29].

Health institutions diagnosing PCa were grouped into private and public. Clinical and treatment variables included diagnostic methods, tumour‐node‐metastasis (TNM) staging, diagnostic prostate‐specific antigen (PSA) level (< 10, 10–20, and > 20 ng/mL), and International Society of Urological Pathology grade group ranging from 1 to 5 (group 1: Gleason score [GS] ≤ 6; group 2: GS 3 + 4; group 3: GS 4 + 3; group 4: GS 4 + 4, 5 + 3 or 3 + 5; group 5: GS 4 + 5, 5 + 4 or 5 + 5) [30]. Risk groups included NCCN high‐risk‐to‐very‐high‐risk (cT3‐T4, GS = 8–10, or PSA > 20 ng/mL), regional (T_any_N_1_M_0_ or node‐positive) and metastatic (T_any_N_any_M_1_) PCa [30]. PCa treatment categories included no treatment (±active surveillance/watchful waiting), surgery, radiation therapy, androgen deprivation therapy (ADT) (±chemotherapy), and other (focal therapies or ablation).

#### 2.4.3 Outcomes

Death data were ascertained through linkage to VCR, which receives biannual death data updates from the Victorian Registry of Births, Deaths, and Marriages. VCR used the tenth version of the International Classifications of Diseases–Australian Modification (ICD‐10‐AM) to code underlying causes of death [20]. The outcomes were ACM and PCSM. ACM encompasses deaths from any cause, whereas PCSM only includes deaths where PCa was the underlying cause.

We calculated survival time from date‐of‐diagnosis to date‐of‐death or last follow‐up (censoring) in months. The final follow‐up date was 31 July 2024. Individuals alive at final follow‐up were censored for ACM. Individuals deceased from causes other than PCa, or alive at final follow‐up, were censored for PCSM.

### 2.5 Statistical Analysis

Relevant descriptive statistics were calculated. For ACM, overall survival (OS) was estimated using the Kaplan–Meier method [31]. We reported median survival time and follow‐up time with 95% confidence intervals (CIs). Overall and cumulative survival percentages (5‐year and 10‐year) with 95% CIs by NCCN risk groups were presented. The log‐rank test assessed differences in OS by NCCN risk group by individuals' CALD status. For PCa‐specific survival (PCSS), we calculated the cumulative incidence function (CIF)—the cumulative probability of PCa death in the presence of competing deaths. CIFs of PCSS with 95% CIs were computed. Differences in CIF across CALD status subgroups were compared using the Gray's test [31].

We fitted two models to examine associations between CALD status and each of ACM and PCSM. Initially, we fitted univariable and multivariable Cox proportional hazards (PH) models for time‐to‐ACM (results not shown). When the PH assumption was checked for covariates using the log–log survival curve and the Schoenfeld residual test, we found that the assumption of constant hazard over time was violated (X^2^ = 134, df = 8, *p* < 0.001). Therefore, we fitted univariable and multivariable generalised Weibull parametric regression models to examine associations between CALD status and time‐to‐ACM. The Weibull parametric survival model is appropriate if time‐to‐event data do not satisfy the PH assumption [31]. The shape parameter (*p* = 1.28) from generalised Weibull parametric regression indicates increasing hazard over time. We also fitted univariable and multivariable Fine‐Gray competing‐risks regression models to investigate associations between CALD status and time to‐PCSM [32]. Accounting for competing risks can provide more accurate estimates for analyses of time to‐disease‐specific events [32].

For each outcome, three multivariable regression models were stepwise‐adjusted for different covariate combinations. Model 1 (minimally adjusted) was adjusted for age (continuous, per 5‐year increment). Model 2 (moderately adjusted) included age, other sociodemographic variables, and diagnosing health institutions. Model 3 (fully adjusted) included all variables in models 1 and 2 plus NCCN risk group and treatment modalities. Stepwise adjustment was made in accordance with previous research [33]. Weibull parametric regression computed a hazard ratio (HR) and 95% CI. Fine‐Gray competing‐risks regression also calculated the subdistribution HR (sHR) with 95% CI.

For each outcome, we investigated whether age and NCCN risk category interact with CALD status. Age and NCCN risk category were included in interaction terms on the basis of prior literature [34, 35]. In particular, age is a crucial consideration in PCa screening recommendations and treatment decisions, and it predicts survival outcomes, which may vary by CALD status [36]. Multiplicative interaction terms between CALD status and each of age at diagnosis and NCCN risk category were added to models separately for each outcome. Marginal predicted hazards for ACM and PCSM were computed and graphed to elucidate interaction effects. Our primary analysis was a complete‐case analysis for key variables.

We also conducted a sensitivity analysis to assess whether missing data on country of birth and NCCN risk category substantially altered key findings of the primary analyses, thereby assessing our assumption that data on these key variables were missing at random (MAR). The missing data pattern was examined using the Little missing completely at random (MCAR) test [37]. A binary logistic regression model was fitted to assess whether missing data on key variables (e.g., country of birth) were associated with observed sociodemographic and clinical data. Multiple imputation using the chained equation model was then applied to observations with missing values; the aforementioned models were refitted to examine the association between CALD status and each of ACM and PCSM. *p*‐values < 0.05 denote statistical significance. Statistical analyses were performed using Stata v17 (StataCorp, College Station, TX).

### 2.6 Ethics Statement

We used deidentified, patient‐level data from PCOR‐Vic. PCOR‐Vic obtained informed consent for participation from all registrants. The present study obtained ethical approval from Monash University Human Research Ethics Committee (ID: 36187).

## 3 Results

### 3.1 Participant Characteristics

Of 49,411 PCOR‐Vic registrants, 22,860 were eligible. Of these, 10,024 participants with high‐risk, regional, or metastatic PCa (44%) were included in the final analysis. The 12,836 exclusions (56%) were due to missing data on NCCN risk group (32%), country of birth (23%), vital status (1%), and other demographic variables (0.1%) (Figure 1).

Data for 10,024 individuals were analysed. Most participants (68%) were born in Australia, 22% were born in NESC (i.e., CALD individuals), and 10% were born in MESC. Median ages at diagnosis (interquartile ranges) were 71 (65–77), 73 (66–79), and 74 (68–80) years for Australian‐born, MESC‐born, and CALD‐background individuals, respectively. More CALD individuals (87%) resided in metropolitan areas than MESC‐born (69%) and Australian‐born (56%) individuals. CALD individuals had a higher proportion of cases diagnosed in public health services (63%) than MESC‐born (53%) and Australian‐born (47%) individuals. Twenty‐eight per cent of the cohort had metastatic PCa at diagnosis: 31% of CALD individuals, 30% of MESC‐born individuals, and 27% of Australian‐born individuals (Table 1).

**TABLE 1:** Sociodemographic and clinical characteristics of individuals diagnosed with high‐risk, regional, or metastatic prostate cancer, overall and stratified by culturally and linguistically diverse status, Victoria, Australia (*N* = 10,024).

Characteristics	Overall (*n* = 10,024)	CALD status		
		Australia‐born (*n* = 6816)	MESC‐born (*n* = 972)	CALD‡‡ (*n* = 2236)
Median (IQR) age at diagnosis (years)	72 (66–78)	71 (65–77)	73 (66–79)	74 (68–80)
Total person‐month follow‐up	551,469	377,623	52,548	121,298
	Frequency, *n* (column %)			
Vital status				
Alive	6816 (68)	4733 (69)	654 (67)	1429 (64)
Died from PCa	1919 (19)	1267 (19)	189 (19)	463 (21)
Died from causes other than PCa	1289 (13)	816 (12)	129 (13)	344 (15)
Causes of death other than PCa				
Non‐cancer causes	790 (8)	499 (7)	74 (8)	217 (10)
Other cancers	322 (3)	198 (3)	40 (4)	84 (4)
Unknown causes	177 (2)	119 (2)	15 (2)	43 (2)
Median survival time for all participants, months (IQR)	116 (49–158)	117 (52–161)	111 (48–172)	105 (43–158)
Median follow‐up time for censored participants, months (IQR)^c^	66 (41–94)	66 (40–93)	64 (41–90)	67 (43–96)
Regions of birth				
Australia	6816 (68)	6816 (100)	—	—
Oceania exc. Australia	194 (2)	—	126 (13)	68 (3)
North‐West Europe	1084 (11)	—	762 (78)	322 (14)
South and Eastern Europe	1118 (11)	—	0 (0)	1118 (50)
North Africa and the Middle East	142 (1)	—	0 (0)	142 (6)
South‐East Asia	183 (2)	—	0 (0)	183 (8)
North‐East Asia	133 (1)	—	0 (0)	133 (6)
South and Central Asia	155 (2)	—	0 (0)	155 (7)
Americas	85 (1)	—	38 (4)	47 (2)
Sub‐Saharan Africa	114 (1)	—	46 (5)	68 (3)
Residential area				
Metropolitan (MM1)	6409 (64)	3790 (56)	670 (69)	1949 (87)
Non‐metropolitan (MM2‐7)	3615 (36)	3026 (44)	302 (31)	287 (13)
SES (SEIFA‐IRSD quintiles)				
Lower	1575 (16)	1101 (16)	112 (11)	362 (16)
Lower‐middle	1290 (13)	1039 (15)	106 (11)	145 (6)
Middle	1842 (18)	1222 (18)	213 (22)	407 (18)
Middle‐upper	1996 (20)	1342 (20)	212 (22)	442 (20)
Upper	3321 (33)	2112 (31)	329 (34)	880 (39)
Diagnosing health institution				
Public	5133 (51)	3207 (47)	516 (53)	1410 (63)
Private	4891 (49)	3609 (53)	456 (47)	826 (37)
Years of diagnosis				
2009–2016	2833 (28)	1.905 (28)	250 (26)	678 (30)
2017–2019	3370 (34)	2285 (33)	346 (36)	739 (33)
2020–2024	3821 (38)	2636 (39)	376 (39)	819 (37)
Diagnostic methods				
Transperineal biopsy	5417 (54)	3756 (55)	524 (54)	1137 (51)
TRUS	25,251 (25)	1759 (26)	224 (23)	542 (24)
TURP	1023 (10)	682 (10)	104 (11)	237 (11)
Other^a^	1058 (11)	618 (9)	120 (12)	320 (14)
NCCN risk group				
High‐risk localised PCa	6400 (64)	4428 (65)	598 (62)	1374 (61)
Regional PCa	841 (8)	581 (8)	86 (9)	174 (8)
Metastatic PCa	2783 (28)	1807 (27)	288 (30)	688 (31)
PSA level				
< 10 ng/mL	3404 (36)	24,694 (38)	280 (31)	655 (31)
10–20 ng/mL	2128 (23)	1411 (22)	213 (23)	504 (24)
> 20 ng/mL	3907 (41)	2561 (40)	421 (46)	925 (44)
Not recorded (missing)^d^	591	381	58	152
Diagnostic Gleason score group				
ISUP 1 (Gleason score ≤ 6)	218 (2)	141 (2)	23 (3)	54 (3)
ISUP 2 (Gleason score 3 + 4)	696 (8)	468 (8)	63 (7)	165 (8)
ISUP 3 (Gleason score 4 + 3)	976 (11)	673 (11)	100 (12)	203 (10)
ISUP 4 (Gleason score 4 + 4) 5 + 3; 3 + 5	2696 (30)	18,540 (30)	264 (31)	578 (30)
ISUP 5 (Gleason score 4 + 5) 5 + 4; 5 + 5	4439 (49)	3095 (50)	407 (47)	937 (48)
Not recorded (missing)^d^	999	585	115	299
T‐stage				
T1	1417 (22)	984 (22)	111 (18)	322 (23)
T2	2233 (34)	1565 (34)	208 (34)	460 (33)
T3	2474 (38)	1722 (38)	238 (39)	514 (37)
T4	434 (6)	295 (6)	51 (8)	88 (6)
Not recorded (missing)^d^	3466	2250	364	852
N‐stage				
0	2297 (23)	5086 (76)	705 (75)	1646 (76)
1	74,335 (74)	1534 (23)	233 (25)	530 (22)
Not recorded (missing)^d^	294	200	34	60
M‐stage				
0	7067 (71)	4894 (72)	665 (69)	1508 (68)
1	2885 (29)	1878 (28)	295 (31)	712 (32)
Not recorded (missing)^d^	72	44	12	16
Treatment modalities				
No treatment	561 (6)	368 (5)	55 (6)	138 (6)
Surgery	3303 (33)	2421 (36)	292 (30)	590 (26)
Radiation	3162 (31)	2138 (31)	299 (31)	725 (32)
ADT (+/−chemotherapy)	2716 (27)	1711 (25)	299 (31)	706 (32)
Other^b^	282 (3)	178 (3)	27 (3)	77 (3)

*Note:* No treatment category includes active surveillance and watchful waiting (AS/WW). NCCN risk calculation: High‐risk‐to‐very‐high‐risk (cT3‐T4, GS = 8–10, or PSA > 20 ng/mL), regional (TanyN1M0 or node‐positive) and metastatic (TanyNanyM1) PCa [30]. CALD‡‡ is defined as being born in non‐English speaking countries. Percentages are rounded and may not add up to 100%.

Abbreviations: ADT, androgen deprivation therapy; CALD, culturally and linguistically diverse; IQR, interquartile range; ISUP, international society of urological pathology; MESC, mainly English‐speaking countries; SEIFA‐IRSD, socioeconomic indexes for areas‐index of socio‐economic disadvantage; PSA, prostate‐specific antigen; TRUS, transrectal ultrasound scan; TRUP, transurethral resection of the prostate.

^a^ Other includes procedures like pathologic tests of metastatic sites.

^b^ Other includes treatment modalities of ablation therapies and treatment not yet specified.

^c^ Median follow‐up time (IQR) for censored participants was estimated using the reverse Kaplan–Meier method.

^d^ When some of PSA, T‐stage, N‐stage, M‐stage, diagnostic Gleason score, or ISUP were not recorded (missing), the NCCN risk category group was determined based on non‐missing data for a smaller subset of the variables PSA, T‐stage, N‐stage, M‐stage, diagnostic Gleason score, and ISUP.

### 3.2 All‐Cause Mortality

#### 3.2.1 Overall Survival

Thirty‐two percent of participants died: 19% from PCa and 13% from other causes. More CALD individuals (10%) died from non‐cancer causes than MESC‐born (8%) and Australian‐born (7%) individuals. Overall, the median (IQR) survival time was 116 months (49–158 months). CALD individuals had shorter median (IQR) survival times (105 [43–158] months) than MESC‐born (111 [48–172] months) and Australian‐born (117 [52–161] months) individuals (Table 1).

At 5‐ and 10‐year follow‐up, there was significant variation in OS by NCCN risk groups and CALD status. At 10‐year follow‐up, CALD individuals with regional PCa had significantly lower OS than Australian‐born individuals with regional PCa (47% vs. 60%) (Table 2).

**TABLE 2:** Overall and prostate cancer‐specific survival probability (%) for the entire cohort, and stratified by individuals' culturally and linguistically diverse status and National Comprehensive Cancer Network risk group, at 5‐year and 10‐year follow‐up among individuals with high‐risk, regional, or metastatic prostate cancer (*N* = 10,024).

CALD status	High‐risk		Regional		Metastatic		All risk groups combined	
	5 years	10 years	5 years	10 years	5 years	10 years	5 years	10 years
Overall survival probability (%, 95% CI)^a^								
Australian‐born individuals	84 (83–85)	69 (67–71)	76 (72–80)	60 (55–65)	40 (37–42)	27 (25–30)	71 (70–72)	55 (54–57)
MESC‐born individuals	82 (79–86)	69 (64–74)	71 (59–80)	55 (41–67)	38 (32–44)	25 (20–31)	67 (64–70)	53 (49–57)
CALD individuals	82 (79–84)	66 (63–69)	63 (54–71)	47 (38–56)	36 (32–40)	24 (20–27)	66 (63–68)	49 (46–51)
All individuals	84 (83–85)	68 (67–70)	73 (69–76)	57 (53–61)	39 (37–41)	26 (24–28)	70 (69–71)	53 (52–55)
*p* ^a^	0.12	0.32	0.26	0.003	0.08	0.11	< 0.001	< 0.001
Prostate cancer‐specific survival probability (%, 95% CI)^b^								
Australian‐born individuals	94 (93–94)	82 (80–84)	88 (8491)	73 (66–79)	53 (50–56)	40 (36–43)	83 (82–83)	70 (69–72)
MESC‐born‐individuals	94 (92–96)	85 (79–90)	82 (70–91)	79 (66–89)	50 (44–57)	38 (29–49)	80 (77–83)	71 (66–76)
CALD individuals	93 (92–95)	84 (80–87)	85 (78–91)	64 (50–78)	53 (49–57)	42 (36–47)	80 (80–82)	70 (67–73)
All individuals	94 (93–94)	83 (81–84)	87 (84–89)	72 (66–77)	53 (51–55)	40 (37–43)	82 (81–83)	70 (69–72)
*p* ^b^	0.50		0.60		0.70		—	

*Note:* Statistically significant when *p* < 0.05.

Abbreviations: CALD, culturally and linguistically diverse backgrounds; CI, confidence interval; MESC, mainly English‐speaking countries.

^a^ Percentage estimated with Kaplan–Meier and log‐rank test method.

^b^ Percentages estimated with the cumulative incidence function and equality across groups checked using the Grey test.

#### 3.2.2 Associations Between Culturally and Linguistically Diverse Status and All‐Cause Mortality

##### 3.2.2.1 Main Effect

In univariable Weibull parametric regression, CALD individuals had a 20% higher hazard of ACM than Australian‐born individuals (crude HR [cHR] = 1.20, 95% = 1.11–1.30). Following adjustment for age, the association between CALD status and ACM was statistically non‐significant (adjusted HR [aHR] = 1.04, 95% CI = 0.95–1.12). Following maximal adjustment for health service type and clinical variables, the direction of association between CALD status and ACM reversed and became statistically significant: CALD individuals had an 11% lower hazard of ACM than Australian‐born individuals (aHR = 0.89, 95% CI = 0.82–0.97) (Table 3).

**TABLE 3:** Results for the univariable and multivariable Weibull parametric regression analyses examining associations between participant characteristics and all‐cause mortality, as well as selected interactions with culturally and linguistically diverse status, among individuals diagnosed with high‐risk, regional, and metastatic prostate cancer in Victoria, Australia, 2009–2024 (*N* = 10,024).

Variables	cHR (95% CI)	Multivariable models, aHR (95% CI)^c^		
		Model 1 (minimal adjustment)	Model 2 (medium adjustment)	Model 3 (maximal adjustment)
Age at diagnosis	1.43 (1.40–1.46)*	1.43 (1.40–1.46)*	1.43 (1.40–1.46)*	1.26 (1.24–1.29)*
CALD status				
Australian‐born individuals	1.00	1.00	1.00	1.00
MESC‐born individuals	1.09 (0.97–1.23)	1.02 (0.90–1.14)	0.97 (0.86–1.10)	0.95 (0.84–1.07)
CALD‡‡ individuals	1.20 (1.11–1.30)*	1.04 (0.95–1.12)	0.90 (0.81–0.98)*	0.89 (0.82–0.97)*
Residential area				
Metropolitan	1.00	—	1.00	1.00
Non‐metropolitan area	0.97 (0.89–1.04)	—	0.89 (0.81–0.98)*	0.91 (0.83–1.00)*
SES (SEIFA IRSD quintile)				
Lower	1.00	—	1.00	1.00
Lower‐middle	0.83 (0.73–0.95)*	—	0.85 (0.75–0.97)*	0.86 (0.76–0.98)*
Middle	0.84 (0.75–0.95)*	—	0.92 (0.81–1.03)	0.94 (0.83–1.06)
Middle‐upper	0.84 (0.75–0.94)*	—	0.93 (0.82–1.05)	0.95 (0.84–1.07)
Upper	0.82 (0.74–0.91)*	—	0.90 (0.80–1.01)	0.91 (0.81–1.02)
Year of diagnosis				
2009–2016	1.00		1.00	1.00
2017–2019	0.90 (0.83–0.97)*		0.81 (0.74–0.87)*	0.80 (0.75–0.87)*
2020–2024	0.87 (0.79–0.96)*		0.78 (0.71–0.87)*	0.76 (0.68–0.84)*
Diagnosing health institution				
Public	1.00	—	1.00	1.00
Private	0.55 (0.51–0.59)*	—	0.54 (0.50–0.59)*	0.77 (0.72–0.83)*
NCCN risk group				
High‐risk	1.00	—	—	1.00
Regional	1.82 (1.59–2.10)*	—	—	1.51 (1.30–1.74)*
Metastatic	4.91 (4.57–5.29)*	—	—	2.85 (2.60–3.11)*
Treatment modalities				
No treatment	1.00	—	—	1.00
Surgery	0.20 (0.17–0.24)*	—	—	0.37 (0.31–0.44)*
Radiation	0.75 (0.64–0.87)*	—	—	0.81 (0.70–0.95)*
ADT (−/+ chemotherapy)	2.29 (1.99–2.64)*	—	—	1.38 (1.18–1.60)*
Other	2.29 (1.86–2.82)*		—	2.31 (1.87–2.85)*
Interaction effect estimates				
Two‐way interaction between CALD status and age at diagnosis				
MESC* Age	—	—	—	1.01 (0.94–1.08)
CALD* Age	—	—	—	1.07 (1.02–1.13)*
Two‐way interaction between CALD and NCCN risk category^a^				
MESC* Regional	—	—	—	1.07 (0.67–1.71)
MESC* Metastatic	—	—	—	1.12 (0.87–1.44)
CALD* Regional	—	—	—	1.50 (1.09–2.08)*
CALD* Metastatic	—	—	—	1.07 (0.90–1.27)
Two‐way interaction between CALD and NCCN risk category^b^				
Australian‐born* High‐risk		—	—	1.00
Australian‐born* Regional	—	—	—	1.36 (1.14–1.63)*
Australian‐born* Metastatic	—	—	—	2.77 (2.50–3.08)*
MESC‐born* High‐risk	—	—	—	0.89 (0.73–1.08)
MESC‐born* Regional	—	—	—	1.30 (0.87–1.94)
MESC‐born* Metastatic	—	—	—	2.78 (2.33–3.30)*
CALD* High‐risk	—	—	—	0.84 (0.73–0.96)*
CALD* Regional	—	—	—	1.72 (1.33–2.23)*
CALD* Metastatic	—	—	—	2.49 (2.19–2.85)*

*Note:* No treatment category may include active surveillance and watchful waiting (AS/WW). CALD‡‡: defined as being born in non‐English speaking countries. Model 1: adjusted for age‐at‐diagnosis (continuous, 5‐year band), Model 2: adjusted for age‐at‐diagnosis (continuous, 5‐year band), residential place, SEIFA‐IRSD quintiles, year of diagnosis, and diagnosing health institution. Model 3: adjusted for age‐at‐diagnosis (continuous, 5‐year band), residential place, SEIFA‐IRSD quintiles, year of diagnosis, diagnosing health institution, NCCN risk group, and treatment modality.

Abbreviations: ADT, androgen deprivation therapy; aHR, adjusted hazard ratio; AS/WW, active surveillance and watchful waiting; CALD, culturally and linguistically diverse backgrounds; CI, confidence interval; cHR, crude hazard ratio; ISUP, international society of urology pathology; MESC, mainly‐English speaking countries; NCCN, national comprehensive cancer network; PSA, prostate‐specific antigen; SEIFA‐IRSD, socioeconomic index for area index of socioeconomic disadvantage.

* Denotes *p*‐value less than 0.05.

^a^ Represents a two‐way interaction effect between CALD and NCCN risk category, with the main effect.

^b^ Represents a two‐way interaction effect between CALD and NCCN risk category, without the main effect.

^c^ Step‐by‐step model adjustments.

##### 3.2.2.2 Interactions

**FIGURE 2:**
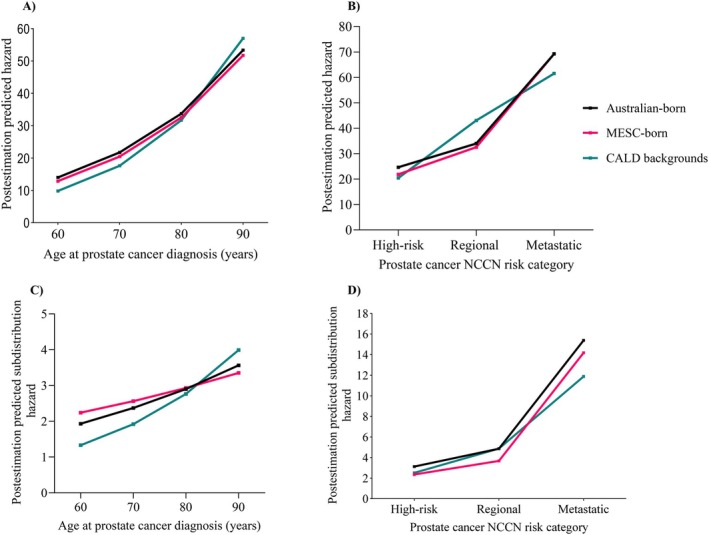
Line graph showing postestimation predicted marginal hazard of all‐cause and prostate cancer‐specific mortality by culturally and linguistically diverse status with other variables constrained at their means: (A) predicted all‐cause mortality by age at diagnosis, (B) predicted all‐cause mortality by prostate cancer National Comprehensive Cancer Network risk category, (C) predicted subdistribution hazard by age at diagnosis, and (D) predicted subdistribution hazard by National Comprehensive Cancer Network risk category. CALD, culturally and linguistically diverse backgrounds; MESC, mainly English‐speaking countries; NCCN, National comprehensive cancer network.

The hazard of ACM changed with interactions between CALD and each of age (global test *p*‐value_interaction_ = 0.015) and NCCN risk category at diagnosis (regional × CALD: *p*‐value = 0.013). Figure 2 illustrates the postestimation predicted hazard of ACM by age, NCCN risk category and CALD status. The hazard of ACM tended to be higher in CALD individuals, particularly those diagnosed at older ages with regional PCa (Table 3; Figure 2).

### 3.3 Prostate Cancer‐Specific Survival

Of 3208 participants who died during follow‐up, 1919 (60%) died from PCa (Table 1). Cumulative PCSS probabilities were 82% (95% CI = 81%–83%) and 70% (95% CI = 69%–72%) at 5 and 10 years, respectively. Compared with their Australian‐born counterparts, CALD individuals with regional PCa had lower PCSS at 5 years (85% vs. 88%) and 10 years (64% vs. 73%) of follow‐up, although these differences were not statistically significant (Table 2).

#### 3.3.1 Associations Between Culturally and Linguistically Diverse Status and Prostate Cancer‐Specific Mortality

##### 3.3.1.1 Main Effect

In the maximally adjusted multivariable Fine‐Gray competing‐risks subdistribution hazard model, CALD individuals had 14% lower hazard of PCSM than Australian‐born individuals (adjusted sHR [asHR] = 0.86, 95% CI = 0.76–0.96) (Table 4).

**TABLE 4:** Results of univariable and multivariable Fine‐Gray competing risk regression analyses examining associations between participant characteristics and prostate cancer‐specific mortality, as well as selected interactions with culturally and linguistically diverse status, among men diagnosed with high‐risk, regional, and metastatic prostate cancer in Victoria, Australia, 2009–2024 (*n* = 10,024).

Variables	Univariable csHR (95% CI)	Multivariable models, asHR (95% CI)^c^		
		Model 1 (minimal adjustment)	Model 2 (medium adjustment)	Model 3 (maximal adjustment)
Age at diagnosis	1.22 (1.18–1.26)*	1.22 (1.19–1.26)*	1.23 (1.19–1.26)*	1.04 (1.01–1.08)*
CALD status				
Australian‐born individuals	1.00	1.00	1.00	1.00
MESC‐born individuals	1.06 (0.91–1.23)	1.01 (0.87–1.18)	0.97 (0.83–1.13)	0.94 (0.80–1.10)
CALD‡‡ individuals	1.10 (0.99–1.22)	1.00 (0.90–1.11)	0.86 (0.77–0.97)*	0.86 (0.76–0.96)*
Residential area				
Metropolitan	1.00	—	1.00	1.00
Non‐metropolitan	0.95 (0.87–1.05)	—	0.93 (0.83–1.05)	0.96 (0.85–1.09)
SES (SEIFA IRSD) quintiles				
Lower	1.00	—	1.00	1.00
Lower‐middle	0.79 (0.66–0.94)*	—	0.82 (0.69–0.98)*	0.81 (0.68–0.97)*
Middle	0.90 (0.77–1.05)	—	0.98 (0.84–1.14)	0.97 (0.80–1.14)
Middle‐upper	0.91 (0.78–1.05)	—	1.01 (0.86–1.18)	1.03 (0.88–1.21)
Upper	0.89 (0.77–1.01)	—	0.99 (0.85–1.16)	0.97 (0.82–1.13)
Year of diagnosis				
2009–2016	1.00	—	1.00	1.00
2017–2019	0.71 (0.64–0.79)*	—	0.66 (0.59–0.73)*	0.66 (0.59–0.73)*
2020–2024	0.56 (0.56–0.64)*	—	0.51 (0.45–0.58)*	0.48 (0.42–0.54)*
Diagnosing health institution				
Public	1.00	—	1.00	1.00
Private	0.57 (0.52–0.62)*	—	0.55 (0.49–0.60)*	0.97 (0.87–1.07)
NCCN risk group				
High‐risk	1.00	—	—	1.00
Regional	2.02 (1.67–2.46)*	—	—	1.63 (1.34–1.98)*
Metastatic	7.51 (6.80–8.28)*	—	—	4.57 (4.03–5.17)*
Treatment modalities				
No treatment	1.00	—	—	1.00
Surgery	0.20 (0.17–0.24)*	—	—	0.57 (0.43–0.76)*
Radiation	0.75 (0.64–0.87)*	—	—	1.32 (1.03–1.69)*
ADT	2.29 (1.99–2.63)*	—	—	1.91 (1.48–2.46)*
Other	2.31 (1.88–2.84)*	—	—	2.33 (1.64–3.30)*
Interaction effect estimates				
Two‐way interaction between CALD and age at diagnosis				
MESC* age	—	—	—	1.01 (0.92–1.10)
CALD* age	—	—	—	1.10 (1.02–1.17)*
Two‐way interaction between CALD and NCCN risk category^a^				
MESC* Regional	—	—	—	1.04 (0.52–2.07)
MESC* Metastatic	—	—	—	1.34 (0.93–1.93)
CALD* Regional	—	—	—	1.26 (0.80–1.99)
CALD* Metastatic	—	—	—	1.03 (0.81–1.32)
Two‐way interaction between CALD and NCCN risk category^b^				
Australian‐born* High‐risk	—	—	—	1.00
Australian‐born* Regional	—	—	—	1.56 (1.23–1.98)*
Australian‐born* Metastatic	—	—	—	4.35 (3.77–5.02)*
MESC‐born* High‐risk	—	—	—	0.76 (0.56–1.02)
MESC‐born* Regional	—	—	—	1.18 (0.66–2.13)
MESC‐born* Metastatic	—	—	—	4.55 (3.63–5.69)*
CALD* High‐risk	—	—	—	0.80 (0.66–0.98)*
CALD* Regional	—	—	—	1.56 (1.07–2.27)*
CALD* Metastatic	—	—	—	3.81 (3.19–4.55)*

*Note:* No treatment category may include active surveillance and watchful waiting (AS/WW). CALD‡‡: defined as being born in non‐English speaking countries. Model 1: adjusted for age‐at‐diagnosis (continuous, 5‐year band). Model 2: adjusted for age‐at‐diagnosis (continuous, 5‐year band), residential place, SEIFA‐IRSD quintiles, year of diagnosis, and diagnosing health institution. Model 3: adjusted for age‐at‐diagnosis (continuous, 5‐year band), residential place, SEIFA‐IRSD quintiles, year of diagnosis, diagnosing health institution, NCCN risk group, and treatment modality.

Abbreviations: ADT, androgen‐deprivation therapy; asHR, adjusted sub‐distribution hazard ratio; CALD, culturally and linguistically diverse backgrounds; CI, confidence interval; csHR, crude sub‐distribution hazard ratio; ISUP, international society of urology pathology; HR, hazard ratio; MESC, mainly‐English speaking countries; NCCN, national comprehensive cancer network; PSA, prostate‐specific antigen.

* Denotes *p*‐value less than 0.05.

^a^ Represents a two‐way interaction effect between CALD and NCCN risk category, with the main effect.

^b^ Represents a two‐way interaction effect between CALD and NCCN risk category, without the main effect.

^c^ Step‐by‐step model adjustments.

##### 3.3.1.2 Interactions

The association between CALD status and PCSM changed with age (CALD individuals: *p*‐value_interaction_ = 0.009) (Table 4). Figure 2 (C and D) shows the predicted subdistribution hazard of PSM by CALD status across age and NCCN risk category. The subdistribution hazard of PCSM exhibited a different trajectory among CALD individuals aged 80+ years, while the predicted sHR for Australian‐born individuals remained stable across ages (Table 4 and Figure 2).

##### 3.3.1.3 Sensitivity Analysis

We examined missing data patterns, and the outcome variable was more or less complete (approximately 1% missing). Substantial missingness was observed for the participants' country of birth (23%) and NCCN risk category (32%). The Little MCAR test was statistically significant (*p*‐value < 0.001), ruling out the possibility that data were MCAR while suggesting that missingness could be related to the observed data. As patients' sociodemographic (e.g., age at diagnosis and residence) and clinical (e.g., treatment modalities) characteristics were associated with missing values for country of birth in a binary logistic regression model, it was reasonable to assume these data were MAR.

Accounting for missingness in country of birth and NCCN risk category using multiple imputation did not substantially alter effect estimates for the associations between CALD status and each of ACM and PCSM. In particular, in the univariate competing‐risks analysis of imputed data, CALD individuals had higher PCSM than Australian‐born individuals (csHR = 1.10, 95% CI: 1.00–1.21), underscoring that improved recording of key CALD‐defining and clinical variables may reduce bias and enhance the study's power. In multivariable analysis, CALD individuals had lower PCSM than Australian‐born individuals (asHR = 0.90, 95% CI: 0.81–1.00), suggesting that the disparity in PCSM by CALD status was explained by baseline differences in clinical and non‐clinical factors, as evidenced by the complete‐case analysis (Tables S1 and S2).

## 4 Discussion

This retrospective cohort study conducted in Victoria, Australia, found that CALD individuals with high‐risk, regional, and metastatic PCa had lower OS and a higher hazard of ACM than Australian‐born individuals in crude analyses. Following adjustments for clinical and non‐clinical variables, CALD individuals had lower ACM and PCSM compared to Australian‐born individuals. We also found that mortality outcomes changed with statistically significant interactions between CALD status and each of age and NCCN risk category at diagnosis. Inequalities in ACM and PCSM disfavouring CALD individuals diagnosed with PCa, particularly at older ages, were observed. A similar pattern in ACM persisted among CALD individuals with regional PCa compared to non‐CALD counterparts.

Although international comparisons are challenging due to varied immigrant populations across countries, our results generally align with studies in comparable countries (e.g., Britain [38], Canada [39], and the US [40, 41]) showing immigrants have lower overall and cancer‐specific mortality than native‐born individuals.

Lower mortality for CALD than Australian‐born individuals does, however, contradict the fact that CALD individuals tend to be diagnosed at older ages and with more advanced PCa than Australian‐born individuals [18]. Adjusting for clinical and non‐clinical variables reversed the direction of associations between CALD and ACM and PCSM, indicating lower mortality among CALD individuals than among Australian‐born individuals. Our results suggest that, if patients have similar age at PCa diagnosis, NCCN risk categories, health service utilisation, and other demographic characteristics, CALD individuals would have lower mortality than Australian‐born individuals. However, CALD individuals continue to experience challenges in accessing early detection and timely diagnosis, presenting with more advanced PCa and treatment delays [16, 17, 18, 42]. In other words, reversal in the direction of association may indicate unequal distributions of baseline risk factors, including the social determinants of health and access barriers, as a key mechanism underlying survival disparities by CALD status. Several Australian Government health equity initiatives have already acknowledged that equitable and optimal cancer outcomes among CALD individuals can only be achieved by addressing systemic and structural factors influencing early detection and access to safe and effective anti‐cancer treatments [12, 13]. Taken together, our study results provide an important reminder that CALD‐associated disparities in survival outcomes should be considered through an intersectional lens, requiring whole‐of‐government and CALD community engagement.

Another potential explanation for the observed differences in survival outcomes by CALD status may be the HME. First, lower mortality among CALD individuals could align with evidence from the HME, which suggests that healthy, educated, and skilled individuals are preferentially selected for immigration [8, 43]. Despite socioeconomic deprivation, communication/language barriers, structural discrimination, and a lack of culturally sensitive healthcare, individuals from CALD backgrounds tend to have better survival than native‐born individuals [9, 40]. For example, a US study found that foreign‐born Hispanics living in ethnic enclaves (i.e., geographically clustered and socially distinct ethnic groups) had better PCSS than their native‐born counterparts [40]. These benefits may be due to improved social support, cultural preservation, and healthier diets, which mediate mortality risk [40, 44]. In addition, Australian, Canadian, British, and US studies showed that immigrants had significantly fewer chronic conditions than native‐born individuals [8, 26]. In Australia, during 2014–15 and 2017–18, 57% of Australian‐born individuals had age‐adjusted chronic health conditions, compared with 47% of overseas‐born individuals [26, 45]. Therefore, lower mortality among CALD individuals in Australia compared to Australian‐born individuals could also reflect the HME.

When interactions between CALD status and age at diagnosis were considered, disparities in mortality outcomes persisted. After adjusting for clinical and non‐clinical variables, ACM and PCSM were higher for CALD individuals diagnosed with PCa at older ages (80+ years) than their non‐CALD counterparts. Ageing is linked with the accumulation of risk factors, poor recognition of cancer symptoms, and healthcare accessibility issues, which lead to worse prognoses [36, 46, 47]. The effect of age on PCSM could be exacerbated by the types and extent of comorbid conditions associated with ageing. An Australian retrospective cohort study demonstrated that higher comorbidity scores and specific comorbidities (e.g., cardiac disease) were independently associated with worse PCSM [48]. However, our study lacks data on comorbidities. Future research should estimate survival outcomes among CALD individuals while accounting for comorbidities and other relevant factors. The impact of ageing on cancer outcomes is already recognised in the Australian Cancer Plan, which recommends addressing barriers to accessing geriatric care [12].

The effect of regional PCa diagnosis on ACM varied between CALD and non‐CALD individuals. This difference may be due to treatment‐related toxicities, as treatments for advanced PCa (e.g., ADT) are associated with life‐threatening cardiovascular complications. Additionally, treatment modalities for regional PCa, including radical prostatectomy with pelvic lymph node dissection, with or without adjuvant therapy, have been shown to improve survival outcomes [49]. However, not all patients with regional PCa have equitable access to treatment; racial minorities and socioeconomically disadvantaged groups are less likely to receive local therapies for regional PCa, which in turn can affect survival outcomes [50]. In this context, our finding of higher ACM among CALD individuals with regional PCa may warrant further research to elucidate the underlying mechanisms, including the roles of cultural, clinical, and health system factors on quality of treatment decisions and patient preferences.

This study may have important implications for individuals from CALD backgrounds, researchers, the health system, and other relevant stakeholders. While people from CALD backgrounds are recognised as a priority population, benchmark estimates (e.g., survival outcomes for individuals with PCa by CALD status) remain scarce. Our results may fill evidence gaps, highlighting the need to address disparities in survival outcomes and contributing factors. For example, the reversal of the association from higher ACM and comparable PCSM in unadjusted models to lower ACM and PCSM in adjusted models can demonstrate how sociodemographic, clinical, and health system factors explain survival outcome disparities by CALD status. Moreover, disparities in survival outcomes among CALD individuals may signal the need to evaluate whether CALD‐focused interventions (e.g., language services) are appropriately tailored and easily accessible to everyone. Taken together, our study results may serve as an indication to renew and strengthen stakeholders' commitments, including the Australian Cancer Plan [12], which targets people from CALD backgrounds with cancer for equity‐focused interventions. Our study has two key strengths. Firstly, the large sample size of 10,024 individuals with PCa provided sufficient power to detect statistically significant associations. Secondly, there was little missing follow‐up data on vital status (1%) because death data were obtained from the Victorian Registry of Births, Deaths, and Marriages.

Our study also has limitations. Fifty‐six percent of eligible PCOR‐Vic registrants were excluded due to missing data on NCCN risk category, country of birth, vital status, and other sociodemographic factors. Although no prior study has reported survival outcomes by NCCN risk category, our results are comparable with data from the Cancer Council Victoria [51]. The characteristics of the CALD population in our study cohort align with previous Australian findings (e.g., most CALDs were metropolitan residents) [52]. Additionally, the proportions of the overseas‐born population and by region of birth in our study were comparable to ABS data for Victoria, Australia [19].

The representativeness of our sample can be considered in terms of case ascertainment and the completeness of data on key variables. Regarding case ascertainment, the data source, PCOR‐Vic, receives notifications from VCR (which completely enumerates cancer cases in Victoria), accounting for 87% of newly diagnosed PCa cases in the State of Victoria. Institutional coverage in PCOR‐Vic is not 100% [53]. Some private health services, urologists, remote hospitals, delayed notifications within health services, and non‐participating health services may be sources of systematic missingness among PCOR‐Vic registrants, leaving some patients unrecorded [20]. This missingness may be related to unobserved provider‐ and health system‐level characteristics (e.g., health service data‐sharing policy) and cannot be explained by the observed data or statistically tested. However, PCOR‐Vic coverage is high and growing, as evidenced by notifications from various health services [53]. In addition, there is no strong justification or evidence for differential clustering of PCa patients by CALD status in non‐participating health institutions. Instead, CALD patients had lower private health insurance and were more likely to be treated in public health services than Australian‐born patients [9, 54]. In addition, compared with Australian‐born people, proportionately more CALD people live in urban areas [52]. In this context, underrepresentation of CALD patients due to incomplete institutional coverage and related issues may be minimal, and associated selection biases may be low to moderate. Regarding the completeness of country of birth data, as country of birth data are missing from VCR due to hospitals only sending VCR as a subset of hospitalisation records containing information related to cancer diagnoses. As a result, PCOR‐Vic's missingness on the country of birth was likely to be MAR. Models using multiple‐imputation data produced effect estimates comparable to complete‐case analysis, with no substantial change in overall findings. However, unlike the results from the complete‐case analysis, the univariate competing‐risks model using imputed data showed that CALD individuals had a higher PCSM than Australian‐born individuals. This underscores the need to improve the recording of key CALD‐defining and clinical variables to minimise bias and enhance the study's power. Clinical registries with high levels of missing data on key clinical and sociodemographic variables, such as PCOR‐Vic, should prioritise capturing more complete data to enable higher‐quality analyses. Missing country of birth reccord is also recognised as a data quality concern in AIHW's cancer data [2].

We defined CALD status using country of birth to capture first‐generation immigrants; however, this definition excludes and underrepresents other CALD subgroups (e.g., second‐ or higher‐generation immigrants) [27]. Defining one's CALD background by aggregating country of birth is simple and allows a statistically calculable minimum cell size while protecting privacy; it may, however, homogenise diversity and average out within‐group differences among individual minority groups. Further disaggregation of CALD background by preferred language spoken can partially overcome such limitations and identify vulnerable populations, as CALD individuals with PCa who speak a language other than English have worse outcomes and poorer experiences [18, 42].

Our study could have been confounded by unmeasured factors associated with both CALD status and mortality, including comorbidities, individual‐level SES, and time since migration. Although PCOR‐Vic previously attempted to derive comorbidity indices from administrative and medical record auditing, such indices showed poor agreement with patients' self‐reported data and did not reflect the patients' actual disease profile [55]. Additionally, immigration‐related factors, including age at migration, acculturation score, years lived in Australia, and language proficiency, are either unavailable or poorly recorded in PCOR‐Vic. As a result, their effects on survival outcomes could not be assessed, underscoring the need for future studies that account for these variables to build a more comprehensive understanding of CALD individuals' experiences and needs.

## 5 Conclusions

Our study reveals CALD individuals with high‐risk, regional, and metastatic PCa experienced lower OS, higher ACM and similar PCSM compared to Australian‐born counterparts. Following adjustment for clinical and non‐clinical factors, ACM and PCSM were lower for CALD individuals than Australian‐born individuals. Older age at PCa diagnosis has amplified CALD‐related disparities; CALD individuals experienced higher hazards of ACM and PCSM than non‐CALD individuals. Additionally, interactions between CALD status and prognostic factors (NCCN risk category) influenced ACM: CALD individuals with regional PCa had higher ACM compared to non‐CALD individuals. Our findings suggest the need to ensure access to early detection and screening for PCa and geriatric healthcare for CALD individuals, with a view to achieving equitable survival outcomes.

## Author Contributions

**Koku Sisay Tamirat:** conceptualization, investigation, writing – original draft, methodology, formal analysis, writing – review and editing, software. **Nathan Papa:** conceptualization, investigation, methodology, validation, software, writing – review and editing, and supervision. **Jeremy Millar:** conceptualization, investigation, methodology, validation, software, writing – review and editing, and supervision. **Eli Ristevski:** conceptualization, investigation, methodology, validation, software, writing – review and editing, and supervision. **Michael James Leach:** conceptualization, investigation, methodology, validation, software, writing – review and editing, and supervision.

## Funding

The authors have nothing to report.

## Conflicts of Interest

The authors declare no conflicts of interest.

## Supporting information


**Table S1:** Results for the univariable and multivariable Weibull parametric regression analyses examining associations between culturally and linguistically diverse status and its interactions and all‐cause mortality using multiple imputation data, Victoria, Australia (*n* = 21,946).
**Table S2:** Results of univariable and multivariable Fine‐Gray competing risk regression analyses examining associations between culturally and linguistically diverse status and its interactions and prostate cancer‐specific mortality using multiple imputation data, Victoria, Australia (*n* = 21,946).

## Data Availability

The data that support the findings of this study are available on request from the corresponding author. The data are not publicly available due to privacy or ethical restrictions.
