# Supplementary material for: Survival of Individuals Diagnosed With High‐Risk, Regional, and Metastatic Prostate Cancer by Culturally and Linguistically Diverse Status: A Retrospective Cohort Study Set in Victoria, Australia
Source: Cancer Med. 2026 Apr 14;15(4):e71779. doi: 10.1002/cam4.71779 (PMC13079425; doi:10.1002/cam4.71779)
Supplement: Supplementary file 1 — Table S1: Results for the univariable and multivariable Weibull parametric regression analyses examining associations between culturally and linguistically diverse status and its interactions and all‐cause mortality using multiple imputation data, Victoria, Australia (n = 21,946). Table S2: Results of univariable and multivariable Fine‐Gray competing risk regression analyses examining associations between culturally and linguistically diverse status and its interactions and prostate cancer‐specific mortality using multiple imputation data, Victoria, Australia (n = 21,946). [file CAM4-15-e71779-s001.docx]

Supplementary Table S1: Results for the univariable and multivariable Weibull parametric regression analyses examining associations between culturally and linguistically diverse status and its interactions and all-cause mortality using multiple imputation data, Victoria, Australia (n=21,946)

|  | Crude HR | aHR | p-value |
| --- | --- | --- | --- |
| CALD status |  |  |  |
| Australian-born | 1.00 | 1.00 |  |
| MESC-born | 1.05 (0.95-1.16) | 0.92 (0.83-1.02) | 0.128 |
| CALD‡‡ backgrounds | 1.17 (1.09-1.26)* | 0.92 (0.85-0.99) | 0.036 |
| Two-way interaction between CALD and age at diagnosis |  |  |  |
| MESC*age |  | 0.99 (0.93-1.06) | 0.911 |
| CALD*age |  | 1.06 (1.02-1.11) | 0.002 |
| Two-way interaction between CALD and age at diagnosis |  |  |  |
| MESC*Regional |  | 1.01 (0.67-1.55) | 0.926 |
| MESC*Metastatic |  | 1.00 (0.82-1.24) | 0.927 |
| CALD*Regional |  | 1.29 (0.98-1.70) | 0.068 |
| CALD*Metastatic |  | 1.04 (0.88-1.24) | 0.597 |

^HR: Hazard Ratio, aHR: adjusted hazard ratio^

^CALD‡‡: defined as being born in non-English speaking countries^

^Model was adjusted for age-at-diagnosis (continuous, 5-year band), residential place, SEIFA-IRSD quintiles, year of diagnosis, diagnosing health institution, NCCN risk group, and treatment modality^

^* denotes p-value less than 0.05^

Supplementary Table S2: Results of univariable and multivariable Fine-Gray competing risk regression analyses examining associations between culturally and linguistically diverse status and its interactions and prostate cancer-specific mortality using multiple imputation data, Victoria, Australia (n=21,946)

| Variables | Crude sHR | asHR | p-value |
| --- | --- | --- | --- |
| CALD status |  |  |  |
| Australian-born | 1.00 | 1.00 |  |
| MESC-born | 0.94 (0.81-1.09) | 0.72 (0.72-1.01) | 0.074 |
| CALD‡‡ backgrounds | 1.10 (1.00-1.21)* | 0.90 (0.81-1.00) | 0.059 |
| Two-way interaction between CALD and age at diagnosis |  |  |  |
| MESC*age |  | 1.00 (0.90-1.12) | 0.884 |
| CALD*age |  | 1.11 (1.03-1.19) | 0.003 |
| Two-way interaction between CALD and age at diagnosis |  |  |  |
| MESC*Regional |  | 0.94 (0.50-1.76) | 0.861 |
| MESC*Metastatic |  | 1.21 (0.87-1.67) | 0.239 |
| CALD*Regional |  | 1.17 (0.74-1.85) | 0.477 |
| CALD*Metastatic |  | 1.11 (0.89-1.40) | 0.311 |

^sHR: subdistribution Hazard Ratio, asHR: adjusted subdistribution hazard ratio^

^CALD‡‡: defined as being born in non-English speaking countries^

^Model was adjusted for age-at-diagnosis (continuous, 5-year band), residential place, SEIFA-IRSD quintiles, year of diagnosis, diagnosing health institution, NCCN risk group, and treatment modality^

^* denotes p-value less than 0.05^
